# VEGF is an autocrine/paracrine neuroprotective factor for injured retinal ganglion neurons

**DOI:** 10.1038/s41598-020-68488-z

**Published:** 2020-07-24

**Authors:** Nicolas Froger, Frédéric Matonti, Christophe Roubeix, Valérie Forster, Ivana Ivkovic, Nadège Brunel, Christophe Baudouin, José-Alain Sahel, Serge Picaud

**Affiliations:** 1 Sorbonne Université, INSERM, CNRS, Institut de La Vision, 17 rue Moreau, 75012 Paris, France; 2 UMS 29 INSERM Plateforme FluExGen UPMC, 75012 Paris, France; 3 CHNO Des Quinze-Vingts, DHU Sight Restore, INSERM-DGOS CIC 1423, 75012 Paris, France; 4 Fondation Ophtalmologique Adolphe de Rothschild, 75020 Paris, France; 5 Department of Ophthalmology, The University of Pittsburgh School of Medicine, Pittsburgh, PA 15213 USA; 6 Centre Monticelli Paradis, 433 bis rue Paradis, 13008 Marseille, France; 7 Aix Marseille Univ, CNRS, INT, Inst Neurosci Timone, 13005 Marseille, France

**Keywords:** Retina, Retinal diseases

## Abstract

Vascular endothelial growth factor-A (VEGF) is the angiogenic factor promoting the pathological neovascularization in age-related macular degeneration (AMD) or diabetic macular edema (DME). Evidences have suggested a neurotrophic and neuroprotective role of VEGF, albeit in retina, cellular mechanisms underlying the VEGF neuroprotection remain elusive. Using purified adult retinal ganglion cells (RGCs) in culture, we demonstrated here that VEGF is released by RGCs themselves to promote their own survival, while VEGF neutralization by specific antibodies or traps drastically reduced the RGC survival. These results indicate an autocrine VEGF neuroprotection on RGCs. In parallel, VEGF produced by mixed retinal cells or by mesenchymal stem cells exerted a paracrine neuroprotection on RGCs. Such neuroprotective effect was obtained using the recombinant VEGF-B, suggesting the involvement of VEGF-R1 pathway in VEGF-elicited RGC survival. Finally, glaucomatous patients injected with VEGF traps (ranibizumab or aflibercept) due to either AMD or DME comorbidity, showed a significant reduction of RGC axon fiber layer thickness, consistent with the plausible reduction of the VEGF autocrine stimulation of RGCs. Our results provide evidence of the autocrine neuroprotective function of VEGF on RGCs is crucially involved to preserve injured RGCs such as in glaucomatous patients.

## Introduction

Vascular endothelium growth factor-A (VEGF) is well known for its role in the promotion of angiogenesis. It belongs to a family composed by four distinct glycoproteins: VEGF-A, refers to VEGF as the founding member^1^, while the other main members are VEGF-B, VEGF-C and VEGF-D^2–4^. Their biological actions are mediated by three different VEGF receptors, which belong to the tyrosine-kinase receptor group: VEGF-Receptor1 (VEGF-R1); VEGF-Receptor2 (VEGF-R2) and VEGF-Receptor3 (VEGF-R3). While VEGF-B stimulates only VEGF-R1, VEGF-A can activate both VEGF-R1 and -R2. Differently, VEGF-C and VEGF-D bind preferentially to VEGF-R3^4^.

Except its pro-angiogenic action, VEGF has displayed protective effects, in particular on neuronal cells as suggested by some studies. Hence, VEGF reduces the glutamate-induced excitotoxicity on hippocampal neurons^5^ and protects these cells against hypoxia^6^. In eye, VEGF was described as anti-apoptotic factor for photoreceptors^7^, as well as for Müller cells^8^ and retinal pigment epithelium cells^9^.

In recent years, anti-VEGF therapies have become important treatments to limit tumor growth and suppress neovascularization in retinal diseases such as age-related macular degeneration (AMD) and diabetic macular edema (DME)^10^. Anti-VEGF therapies for eye diseases have clear-cut medical benefits, resulting in the regression of neovessels and improvement of visual acuity^11,12^. However, recent data suggest that anti-VEGF therapies may alter the thickness of the nerve fiber layer, which is composed of retinal ganglion cell (RGC) axons on their way to the optic nerve^13^. This negative effect of anti-VEGF therapies on RGCs is consistent with recent reports on the role of VEGF in the neuroprotection of RGCs in animal models of retinal ischemia^14^, optic nerve transection^15^, and glaucoma^16^. However, the cellular mechanisms of VEGF-mediated neuroprotection on RGCs are still unclear, including whether it acts directly or indirectly on RGCs. Here, we provide evidence that VEGF can directly affect RGC survival by activating their VEGF receptors in an autocrine/paracrine mode.

## Results

### VEGF is a paracrine neuroprotective factor for RGCs

We previously showed that conditioned medium isolated from mixed retinal cell cultures (CM-M) can promote the survival of enriched adult rat RGCs in cultures^17^. This effect occurred either in the presence or in the absence of B27 supplement and persisted until 21 days in vitro (DIV) (Fig. S1). We identified the neuroprotective factors contained in this CM-M using the Luminex assay for rat trophic factors. All samples (n = 4) of CM-M contained a large amount of VEGF-A_164_, but no other factors known to promote RGC survival (*i.e.* BDNF, G-CSF, and GM-CSF; Table 1). The presence of VEGF was confirmed and quantified by ELISA technique (106.1 ± 22.6 pg/ml, mean ± SEM, n = 9).

**Table 1:** Growth factors in conditioned medium harvested from mixed retinal cultures.

	Growth factors (pg/ml)			
	VEGF	BDNF	G-CSF	GM-CSF
Control medium	ND	ND	ND	ND
CM-M	**86.8 ± 13.2 (4/4)**	ND	ND	ND

Growth factors were measured in control and conditioned medium harvested from mixed retinal cultures (CM-M). VEGF concentration, expressed as pg per ml, is the mean ± SEM from independent preparations (n = 4), all showing detectable VEGF levels (4/4). ND, no-detectable; VEGF, vascular growth factor; BDNF, brain derived growth factor; G-CSF, granulocyte colony-stimulating factor; MG-CSF, granulocyte-macrophage colony-stimulating factor.

Mixed retinal cultures are composed of a monolayer of macroglial and microglial cells, upon which are found retinal neurons (mainly cone photoreceptors and bipolar cells; Fig. S2). We attributed the origin of VEGF synthesis to microglial cells because these purified cells can produce substantial levels of VEGF in most cultures after only 48 h (17.8 ± 21.7 pg/ml; n = 22; Fig. S3). P urified macroglial cells (including Müller glial cells) did not produce VEGF (Fig. S3), in contrast to results reported in previous studies on isolated Müller cells^18^ or a human Müller cell line (MIO-M1)^9^.

**Figure 1:**
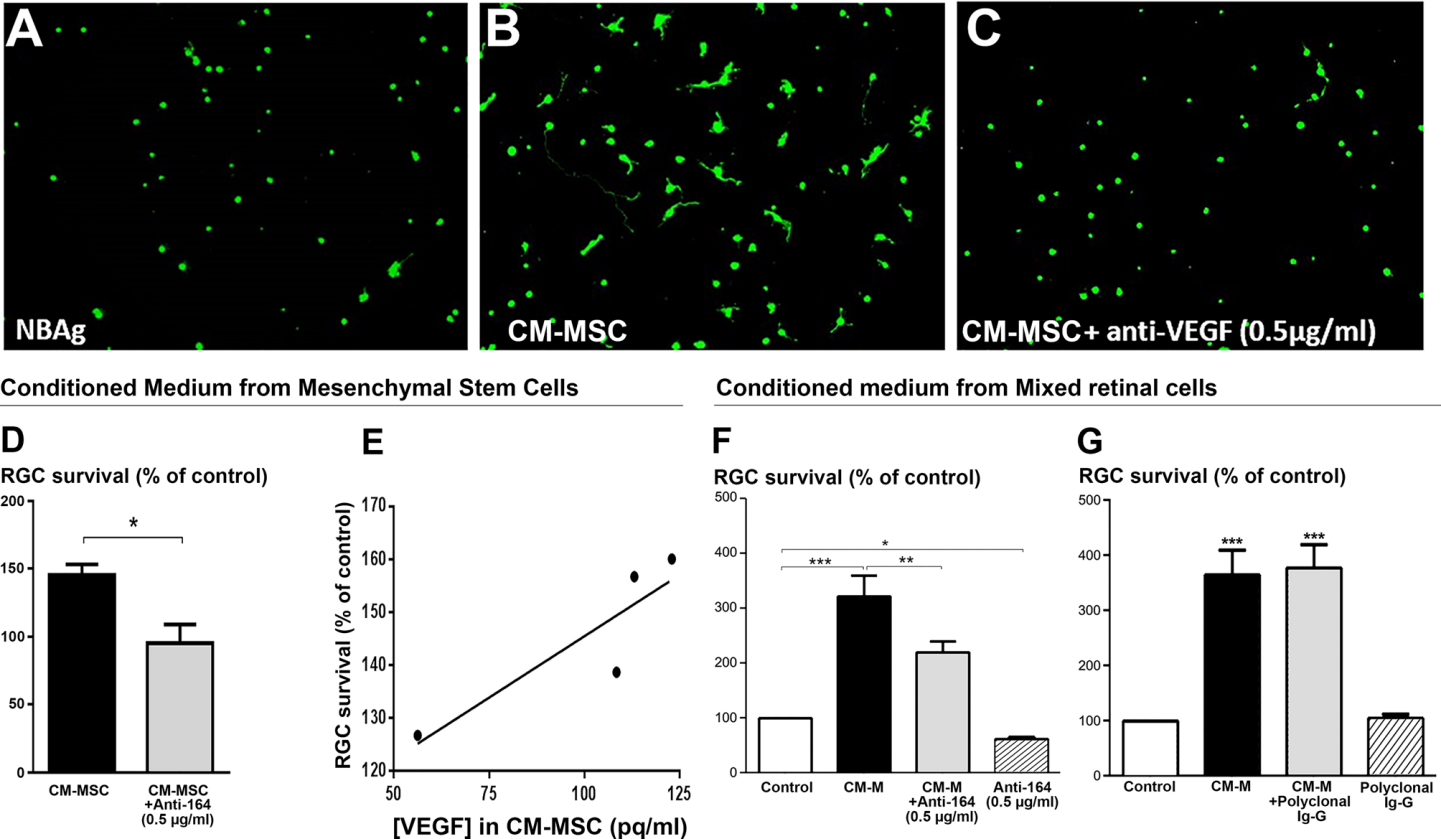
VEGF-elicited survival of retinal ganglion cells (RGCs). (**A**–**C**) Calcein-positive RGCs cultured for 6 DIV in control medium (**A**), conditioned medium from mesenchymal stem cells (CM-MSC) (**B**), or CM-MSC containing a rabbit polyclonal anti-VEGF-A_164_ antibody (**C**). (**D**) RGC survival in CM-MSC, with or without the rabbit polyclonal anti-VEGF-A_164_ antibody. (**E**) Linear correlation between VEGF-A_164_ concentration in CM-MSC and RGC survival. Each point corresponds to an independent experiment. (**F**) RGC survival in the control medium, in conditioned medium from mixed retinal cells (CM-M), in CM-M plus the anti-VEGF antibody, or in control medium plus the anti-VEGF antibody alone. (**G**) Survival of RGCs cultured in control medium, CM-M, CM-M plus an anti-NF200 antibody, or control medium plus the anti-NF200 antibody. Data (means ± SEM) are normalized to the control condition in independent cultures (n = 4 in **D**, **E**; n = 11 in **F**; n = 6 in **G**). RGCs were seeded at initial density of 8000 cells/well. ***p < 0.001, **p < 0.01, and *p < 0.05 as compared to the control group, and ^##^*p* < 0.01 compared between indicated groups (Kruskal–Wallis ANOVA followed by a Dunns *post-hoc* test).

In parallel, we found that conditioned medium from mesenchymal stem cells (CM-MSC), which contained VEGF, can also promote RGC survival (Fig. 1A–E). We assessed whether the VEGF contained in these two different conditioned media (CM-MSC and CM-M) contributed to RGC survival. First, purified adult rat RGCs were incubated with the CM-MSC, in the presence of a specific antibody against VEGF-A_164_ (rabbit polyclonal IgG, 0.5 µg/ml). This anti-VEGF antibody completely suppressed the RGC survival effect produced by the CM-MSC (*p* < 0.01, Fig. 1C,D). Indeed, there was a linear correlation (r^2^ = 0.78) between the VEGF concentration in each CM-MSC and its efficiency in promoting RGC survival (Fig. 1E). Secondly, we obtained a significant reduction of the CM-M-induced neuroprotection (*p* < 0.01) when the anti-VEGF antibody was added to retinal CM-M (Fig. 1F). However, the neuroprotective effect of CM-M was not completely suppressed and remained significant (*p* < 0.01 as compared to control) in presence of the anti-VEGF. This observation may result from the presence of other neurotrophins in CM-M (*e.g.* NGF or GDNF, other than BDNF which were undetectable in CM-M) since the detection of growth factors via our Luminex assay was not exhaustive. Finally, we tested a control rabbit polyclonal IgG targeting another protein (anti-NF200), and we found that the CM-M-enhanced RGC survival was not significantly affected (Fig. 1G). This result indicates that the VEGF present in the two different conditioned media is partially responsible for the trophic effects on RGCs.

### VEGF is an autocrine factor produced by RGCs to promote their own survival

Surprisingly, the antibody directed against VEGF-A_164_ has also reduced per se the survival of purified RGCs by 34.8% when they were cultured in a commercial VEGF-free culture medium (*p* < 0.05, see Fig. 1F). This result suggests that RGCs themselves can release VEGF into culture medium. Indeed, when RGCs were initially seeded at 8000 cells per well, measurable concentrations of VEGF-A_164_ were detected in the culture medium at 6 and 12 DIV by ELISA, whereas VEGF was non-detectable at 1 DIV (not shown). From 70 to 75% of harvested supernatants contained detectable VEGF-A_164_ after 6 to 12 DIV, with concentrations reaching 81.3 ± 12.7 pg/ml at 6 DIV.

**Figure 2:**
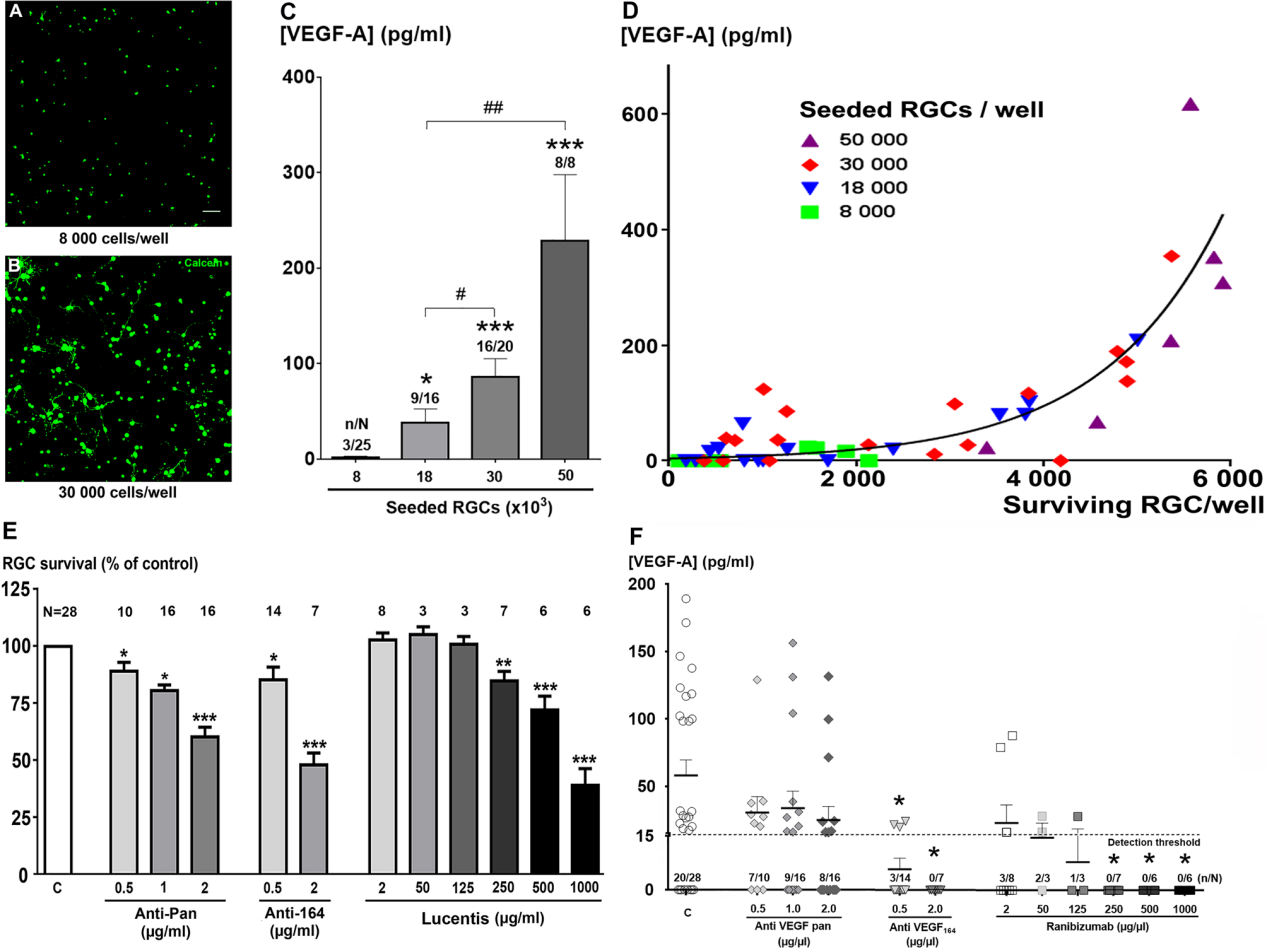
Autocrine VEGF release by retinal ganglion cells (RGCs). (**A**,**B**) Representative confocal images showing live calcein-positive RGCs (6 DIV) with an initial low seeding density (8000 cells/well; **A**) vs. high density (30,000 cells/well; **B**). Scale bar represents 50 µm. (**C**) VEGF-A_164_ concentrations in supernatants from cultured RGCs after 6 DIV, depending on the seeding density. Data are expressed as pg/ml and the means ± SEM from independent cultures with n/N referring to the number of experiments with detectable VEGF A_164_ (n) over the total number of experiments (N). *p < 0.05, **p < 0.01 and ***p < 0.001 as compared to the seeding density of 8 × 10^3^; ^#^*p* < 0.05 and ^##^*p* < 0.01 between indicated groups (Kruskal–Wallis ANOVA followed by the Dunn’s post-hoc test). (**D**) Correlation between the measured VEGF-A_164_ concentration and the actual number of surviving RGCs. The initial seeding densities were 8000 cells/well (green, n = 22), 18,000 cells/well (blue, n = 15), 30,000 cells/well (red, n = 15) or 50,000 cells/well (purple, n = 5). The non-linear regression curve is fitted with an exponential growth equation (r^2^ = 0.65) and a significant correlation was evidenced (*p* < 0.001, Spearman test). (**E**) Suppression of RGC neuroprotection by VEGF-A traps added into the control culture medium: non-selective anti VEGF-A (pan antibody for VEGF isoforms), selective anti-VEGF-A_164_ antibody, and the anti-VEGF Fab fragment ranibizumab. N refers to the total number of experiments in each group. (**F**) VEGF-A_164_ concentrations (in pg/ml) in supernatants from RGC cultures (6 DIV) in the presence of various concentrations of three VEGF traps: anti-pan VEGF-A, anti-VEGF-A_164_, or the VEGF trap ranibizumab. n/N refers to the number of experiments with detectable VEGF A164 (n) over the total number of experiments (N). *p < 0.05 as compared to the control (C) group (Kruskal–Wallis ANOVA followed by the Dunn’s *post-hoc* test). RGCs were seeded at initial density of 30,000 cells/ well in **E**,**F**.

At 6 DIV, the increase of seeding density from 8 × 10^3^ to 50 × 10^3^ cells/well improved the RGC survival (Fig. 2A,B) and increased the percentage of VEGF-positive supernatants from 12 to 100%, respectively (Fig. 2C). VEGF concentrations also substantially and significantly increased with higher seeding density (*p* < 0.001, Kruskal–Wallis test; Fig. 2C). More importantly, the VEGF-A_164_ concentrations highly correlated with the actual number of surviving RGCs (*p* < 0.001, Spearman test), the curve being best fitted with an exponential regression (r^2^ = 0.65) (Fig. 2D). These measures are therefore consistent with a correlation between the level of free VEGF (0-600 pg/ml) and RGC survival (0–6000 RGCs per well). The expression of VEGF in healthy RGCs as in cultured RGCs was confirmed by anti-VEGF immunolabelling of retinal sections and purified RGC cultures (Fig. S4) in agreement with previous reports^9, 19^. These measures are also consistent with the detection of VEGF-A mRNA in embryonic RGCs^20^ and our adult rat RGCs (data not shown). Altogether, these results indicate that VEGF is an autocrine factor for RGCs.

The importance of VEGF for RGC survival was finally verified using various VEGF trapping molecules: (i) a mouse monoclonal anti-human VEGF (anti-pan, non-selective for VEGF-A isoforms), (ii) a rabbit polyclonal antibody against murine VEGF-A_164_ (*i.e.* selective for isoform 164), and (iii) ranibizumab (the Fab fragment from a humanized mouse monoclonal anti-VEGF-A, non-selective for isoforms). These anti-VEGF proteins all induced a statistically significant dose-dependent reduction of RGC survival after 6 DIV (Fig. 2E). The efficacy of ranibizumab became significant at relatively high concentrations (above 250 µg/ml; Fig. 2E). Both the specific rabbit polyclonal antibody against murine VEGF-A_164_ (2 µg/ml), as well as ranibizumab (at least above 250 µg/ml), prevented the detection of VEGF produced by RGCs since 100% of samples displayed undetectable VEFG levels (Fig. 2F). In contrast, the pan antibody (non-selective for VEGF-A isoforms) reduced the proportion of samples with a detectable level of VEGF but did not significantly affect the mean VEGF concentrations in the medium, although it affected its neuroprotective effectiveness, probably due to the reduction of free VEGF (not bound to an antibody). The mean values of VEGF concentrations were calculated, including undetectable values defined as zero. We found that if anti-pan did not significantly decrease the VEGF concentrations whatever the doses used (0.5, 1. or 2.0 µg/ml), the selective anti-VEGF-A_164_ induced a significant decrease in VEGF concentrations in the culture medium at the two doses tested (0.5 and 2.0 µg/ml, *p* < 0.05 as compared to control condition). Similarly, ranibizumab induced a dose-dependent decrease of VEGF concentrations in culture medium, which appeared statistically significant at the dose of 250, 500 and 1,000 µg/ml, respectively (*p* < 0.05 as compared to control condition).

### VEGF-elicited RGC survival involves VEGF-R1 pathway

**Figure 3:**
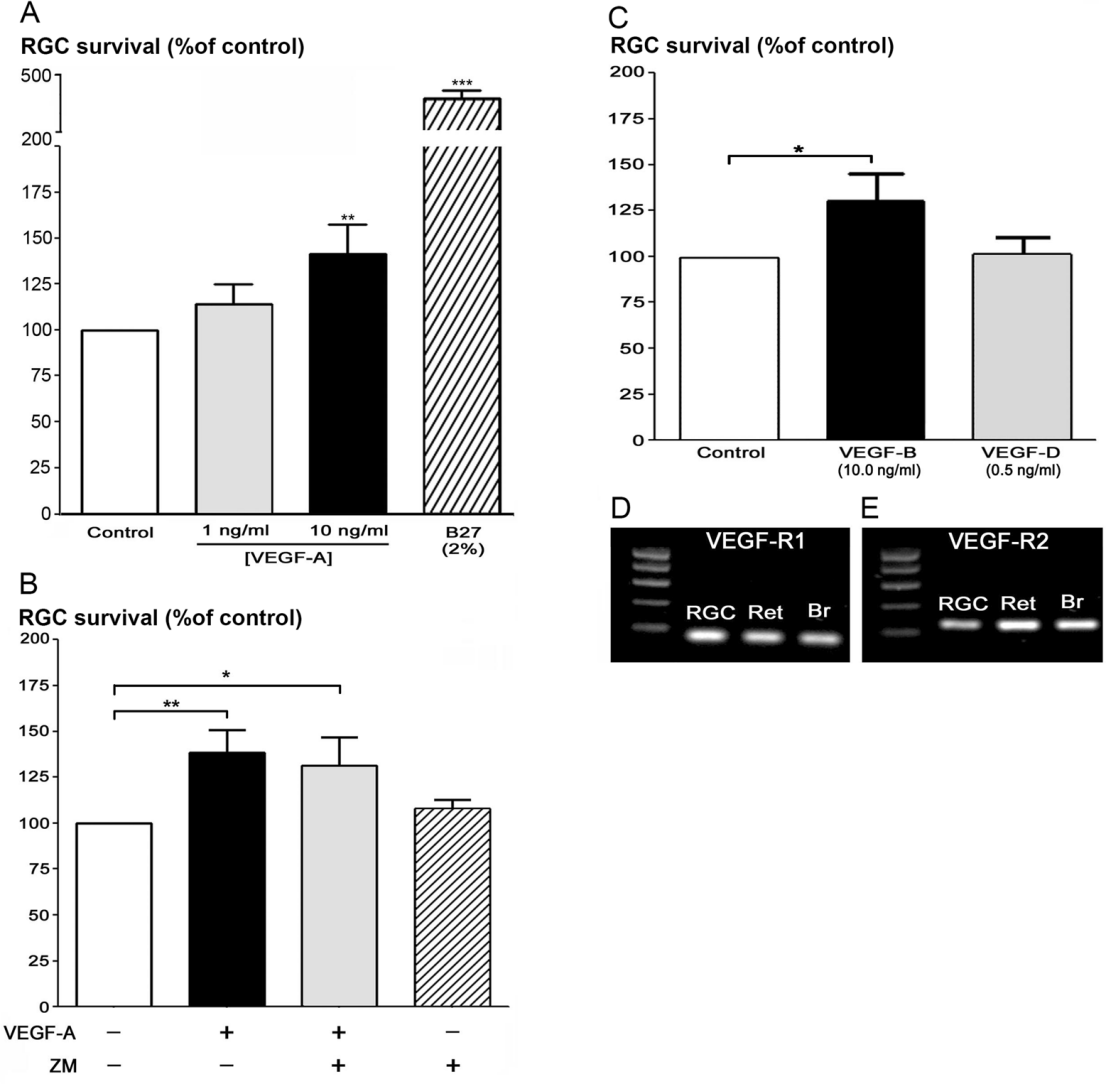
Recombinant VEGF-A_164_ directly stimulates in vitro the RGC survival through VEGF-R1 activation. (**A**) Quantification, by automatic counting, of densities of calcein-positive RGCs cultured for 6 DIV either in the low-nutritive condition (Control; white bar), or with application of two concentration of VEGF-A (1 ng/ml; grey bar and 10 ng/ml, black bar), or with application of the B27 supplement (2%, hatched bar), taken as positive control. (**B**) Quantification of densities of calcein-positive RGCs cultured for 6 DIV either in the low-nutritive condition (Control; white bar), or with application of 10 ng/ml VEGF (black bar), or with 10 ng/ml VEGF plus 5 nM ZM 323,881, a selective VEGF-R2 antagonist (ZM, grey bar) or with 5 nM of ZM 323,881 alone (oblique hatched bar). (**C**) Graph representing the densities of alive calcein-positive RGC cultured for 6 DIV either in the low-nutritive control condition (Control; white bar), or with application of 10 ng/ml VEGF-B (black bar) or 0.5 ng/ml VEGF-D (grey bar). (**D**,**E**) Gene amplification (PCR) of VEGF-Receptor1 (VEGF-R1, **C**) and VEGF-Receptor2 (VEGF-R2; **D**) performed on total cDNAs, previously obtained through a reverse transcription (RT) of total RNA extracted from freshly purified rat retinal ganglion cells (RGC) and rat full retina (Ret), whereas RNA from rat brain tissue (Br), was taken as positive control. These data revealed a high expression of these two-receptor transcripts in enriched RGCs. For each experiment, RGCs were seeded at initial density of 8000 cells/well. The respective RGC densities at 6 DIV were expressed as a percentage of the control. Data are means ± SEM from independent cultures (n = 20 in A, n = 18 in B, n = 11 in C). ***p < 0.001, **p < 0.01 and *p < 0.05 as compared to control (Kruskal–Wallis ANOVA, followed by a Dunns *post-hoc* test).

Using the recombinant VEGF-A_164_ protein (from rat), we consolidated that VEGF per se promotes the RGC survival. At the 10 ng/ml concentration the recombinant protein significantly increased the RGC survival (+ 41.8%, *p* < 0.01), whereas incubation at 1 ng/ml produced a slight increase (+ 14.3%, Fig. 3A), but not statistically significant. To further assess the pharmacological pathway of the VEGF-elicited RGC survival, we investigated the involvement of VEGF receptors in VEGF-elicited RGC survival. Because VEGF-A binds at two distinct receptors named VEGF-R1 and VEGF-R2^21^, we used pharmacological agents in order to discriminate which of these receptors are activated by VEGF to promote the RGC survival. The VEGF-R2 receptor antagonist, ZM 323,881 (ZM) allows discerning the activity of VEGF-R1 versus -R2 receptors. Co application of ZM (5 nM) in the culture medium with the recombinant VEGF-A (10 ng/ml) did not affect the VEGF-mediated increase in RGC survival (ZM + VEGF-A: + 31.4%, VEGF-A: + 38.5%; Fig. 3B), while incubation of ZM alone did not show any significant changes on RGC survival (+ 8.0%). These data suggested that the RGC survival is rather mediated through the VEGF-R1 activation. To verify this hypothesis, we applied a recombinant VEFG-B protein, which stimulates selectively the VEGF-R1. Addition of VEGF-B (1 ng/ml) into the culture medium for 6 DIV significantly increased RGC survival (+ 30.5%; Fig. 3C). In order to check the absence of VEGF-R3 implication in this neuroprotective efficacy, we finally applied the recombinant VEGF-D protein, which activates VEGF-R3 and in some proportion VEGF-R2 (4). However, VEGF-D (0.5 ng/ml) failed to increase the RGC survival (+ 1.7%; Fig. 3C). These results are consistent with the involvement of VEGF-R1 activation by VEGF, to promote RGC survival by VEGF-A. We could show expression of both VEGF-R1 and VEGF-R2—at the mRNA level—in freshly purified RGCs (n = 3), as shown by RT-PCR experiments (Fig. 3D,E). We concluded that RGC survival relies on the VEGF-R1 activation.

### Anti-VEGF treatments alter the retinal nerve fiber layer (RNFL) thickness in glaucomatous patients

**Figure 4:**
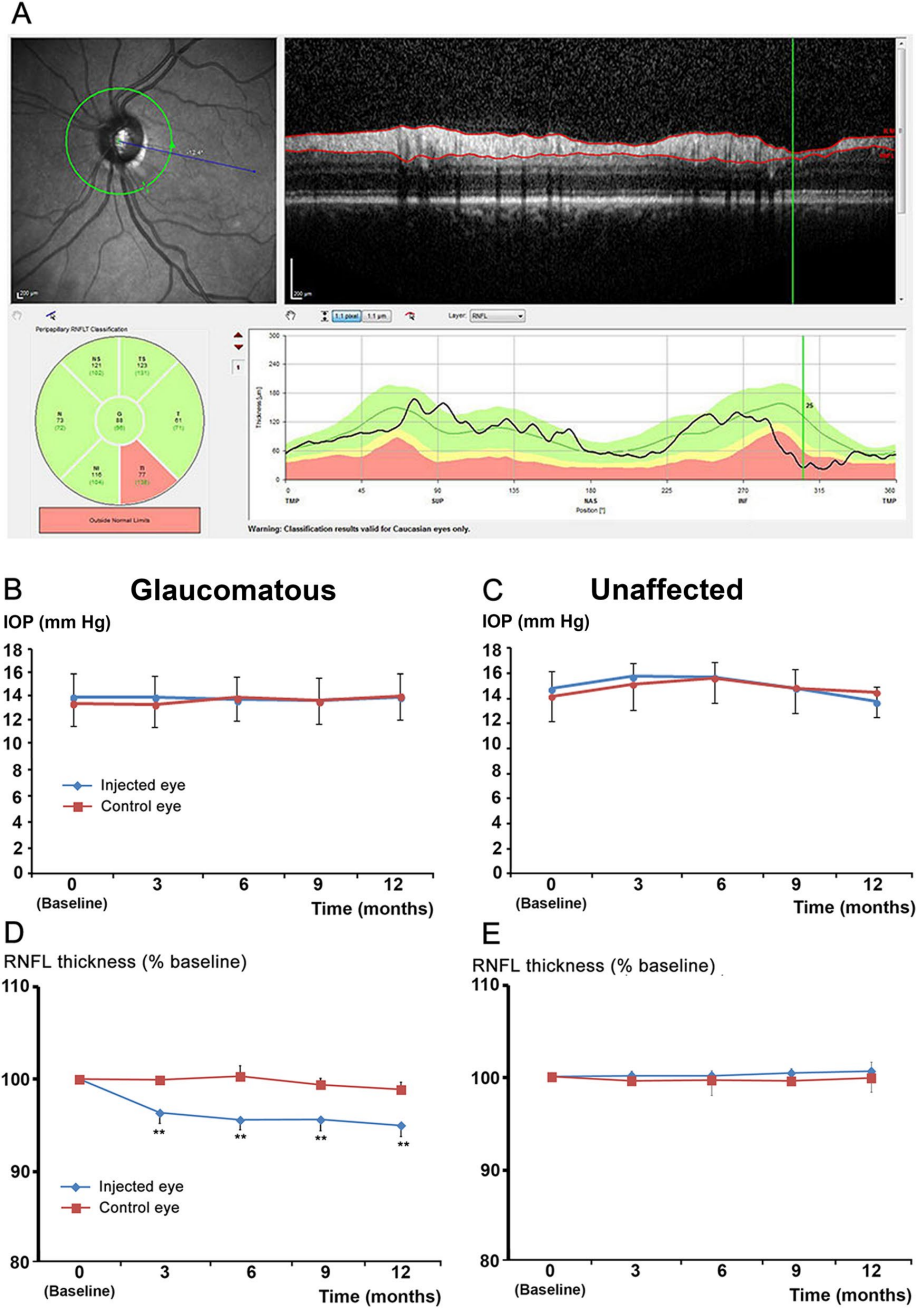
Treatment with VEGF trap reduces RNFL thickness in glaucomatous patients. (**A**) Circular peripapillary OCT scan analysis: An abnormal left eye with its corresponding fundus image (upper left). Red lines in the OCT B-Scan (upper right) indicate the inner and outer borders of the RNFL found by the algorithm. RNFL thicknesses (lower right) plotted over the thickness values measured in healthy subjects of the same age. Mean thickness of the RNFL in the six sectors (lower left). This scan shows a significant decrease (red segment) in the inferior temporal quadrant. B-C: IOP levels in eyes injected with VEGF traps (blue curves, n = 10) or non-injected control eyes (red curves, n = 10) from glaucomatous (**B**) or non-glaucomatous patients (**C**). D-E: RNFL thickness of eyes injected with VEGF trap (blue curves, n = 10) or non-injected control eyes (red curves, n = 10) from glaucomatous (**D**) or non-glaucomatous patients (**E**). **p < 0.01 compared to the baseline value in the treated group (Friedman test followed by a Dunn’s post hoc test).

One report has already suggested that anti-VEGF therapy could negatively affect RGC survival^13^. Such negative effects could be more important in patients with already compromised RGCs. Thus, we focused our investigations by measuring the RNFL thickness of glaucomatous patients, with either AMD or DME comorbidity, as compared to non-glaucomatous patients, all receiving anti-VEGF therapy. There was a statistically significant decrease of RNFL thickness in the treated eyes (injected eyes) from the glaucomatous group, which appeared during the third month of treatment (Fig. 4D). We can observe a quick decrease of the RNFL thickness during the first 3 months corresponding to the induction phase (one intravitreal injection [IVI] each month) whereas during the next phase the decrease is continuing, but less intensely (but remaining significant), probably due to a less intensive treatment (3.4 and 3.2 IVI respectively for non-glaucomatous and glaucomatous patients during the following 9 months).

In contrast, the RNFL thickness of the fellow control eye was stable (Fig. 4D). This decrease was not associated with significant changes in intra-ocular pressure (IOP) levels in either the injected or control eyes (Fig. 4B). There were no differences in RNFL thickness or IOP in either eye of the control group (non-glaucomatous patients; Fig. 4C,E). These observations suggest that anti-VEGF therapies could affect RGC survival in patients with already injured RGCs.

## Discussion

This study provides evidence that VEGF is a neuroprotective factor for RGCs through an autocrine mechanism. We demonstrate in vitro that enriched adult rat RGCs cultured under serum-deprivation—a situation that mimics ischemic conditions—can release VEGF to promote their own survival. To our knowledge, such ability of RGCs to produce themselves VEGF has never been described yet for these retinal neurons, except in one study showing detectable VEGF amounts in supernatant harvested from RGC-5 cell line^22^; the nature of this cell line remaining controversial. Hence, this autocrine mechanism of neuroprotection may underlie the VEGF-elicited neuroprotection of RGCs, as observed in vivo following optic nerve transection^15^ and in glaucomatous animal models^16^. In retina, such neuroprotective action of VEGF involving an autocrine mechanism was already described for Müller cell line (MIO-M1) and retinal pigment epithelium (ARPE-19) which can secrete VEGF to promote their own survival^8,9^. An autocrine mechanism was also suggested to explain the neuroprotective action of VEGF on embryonic cortical neurons^23^, and may be also involved in the VEGF neuroprotection of motoneurons^24^.

In addition, we found that VEGF is released by microglial cells suggesting that it also exerts neuroprotection through paracrine pathway. Other studies attributed the paracrine action of VEGF through a secretion from retinal pigment epithelium (RPE) or from Müller cells. Indeed, VEGF is particularly expressed by RPE^25^ and was also found in inner retina (see Fig. 4S). In human retina, VEGF immunoreactivity was specifically expressed in Müller cell bodies^26^. Moreover, we demonstrate here that VEGF is among the essential factors of MSC secretome conferring a direct RGC protection. A paracrine action of VEGF may also explain part of the therapeutic effect provided by bone marrow-derived MSCs injected into eyes following optic nerve injury or glaucoma^27,28^.

Our pharmacological investigations reveal that VEGF-elicited RGC survival is mediated through the VEGF-R1 stimulation. Indeed, VEGF neuroprotection was not blocked by the addition of VEGF-R2 antagonist, while it was reproduced by incubation of VEGF-B which only stimulates VEGF-R1 (see^4^). VEGF-B was shown to stimulate the survival of RGC-5 subjected to oxidative-stress, and it inhibits the apoptosis in the retina induced by axotomy, NMDA intra-vitreous injection or ischemia^29^, suggesting the neuroprotective effect of the VEGF-R1 signaling. On the other hand, it has been shown that VEGF can protect hippocampal neurons against glutamate excitotoxicity through the activation of VEGF-R2 signaling^5,6^. Furthermore, VEGF-R2 blockade aggravates neuronal degeneration in brain^22,30^, supporting the involvement of VEGF-R2 in VEGF neuroprotection. VEGF-R1 and VEGF-R2 belong to the tyrosine kinase receptor family and both trigger the PI3K/AKT and the MEK/ERK pathways involved in the VEGF neuroprotection^5^. Accordingly, VEGF-R1 and VEGF-R2 can be differentially implied in the neuroprotective effects of VEGF according to the cell types, but in RGCs, VEGF-R1 seems to be preferentially implied.

VEGF production and release by adult rat RGCs demonstrate its role as an autocrine factor for these spiking retinal neurons. In vivo, VEGF is highly expressed by healthy RGCs as observed here by immunolabeling on retinal sections, while a lower expression is found in other cell types (Fig. S4). VEGF release could play a major physiological role because the extracellular space is small and RGC density is very high in the RGC layer. During development, the release of VEGF by RGCs may contribute to the development of the inner retinal vasculature^20^.

We previously showed that cultured RGCs in low nutritive conditions (without B27 serum) have features highly reminiscent of ischemic conditions^17^. Therefore, such culture conditions can be considered as representative of glaucomatous ischemic conditions. As a consequence, these results suggest that the VEGF synthesis is also occurring in RGCs from glaucomatous patients. However, VEGF immunolabeling also demonstrated VEGF synthesis in healthy RGCs^14,15^. Although VEGF concentrations in cell cultures and vitreous are difficult to compare, it is surprising to note that VEGF concentrations released by our cultured RGCs were in a similar range or higher (< 600 pg/ml) than those measured in vitreous of control patients (< 150 pg/ml)^31–33^. It remains unclear if retinal pathologies affecting RGC survival like glaucoma alter VEGF release, thus participating in the dysregulation of ocular blood flow^34^. Apart from its effect on blood flow, reduced VEGF release due to RGC loss could affect the survival of the remaining RGCs in a vicious cycle.

In fact, VEGF contents in the vitreous of glaucomatous patients were only measured in a recent study on patients affected by neovascular glaucoma, showing vitreous VEGF concentrations raising to 220 pg/ml^35^. VEGF contents were higher in vitreous from patients affected by retinal neovascularization, like proliferative diabetic retinopathy, going up to 500 pg/ml, and more (> 1000 pg/ml) according to various studies^31–33,36^. Interestingly, anti-VEGF treatment allowed to drastically reduce the VEGF concentrations in vitreous of these patients^33^. Regarding the healthy subjects, the vitreous VEGF concentrations do not exceed 150 pg/ml (from 1.7 to 146 pg/ml). Although, these VEGF concentrations are in the same range or below those measured in RGC culture media (up to 600 pg/ml), it is unlikely that RGCs provide the only source of VEGF. Indeed, we showed that microglial cells can also synthesize VEGF (Fig. S3) and we previously confirmed other studies showing VEGF production by RPE cells^37^. In addition, others reported that a Müller cell line can also secrete VEGF amounts reaching more than 1000 pg/ml^9^. Therefore, vitreous VEGF is likely resulting from the production by all these retinal cells in vivo*.*

The chronic administration of anti-VEGF therapies may enhance VEGF loss, leading to deleterious consequences for vulnerable RGCs, such as in glaucoma. This safety concern for anti-VEGF therapies is consistent with a previous study showing a significant loss of RNFL thickness after 12 months of intravitreal anti-VEGF injections in AMD patients^13^. This conclusion is further supported by a recent retrospective study showing that patients with continuous unilateral anti-VEGF treatment showed a significant decrease in both RNFL and RGC layer thickness during at least 24 months of follow-up^38^. However, other studies in patients with only AMD have not found any correlation between RNFL changes and anti-VEGF therapy^39–41^. In a recent study^42^ which looked at patients with both glaucoma and AMD, the authors did not find a correlation between decrease nerve fiber layer and treatment. However, the main concern with this study is that they used a macular analysis of ganglion cells or RNFL, and the macular edema due to AMD may induce artifacts on the measures of thickness for each macular layer (ganglion cells, RNFL) with artificial increase, thus probably masking a thinning of these layers. Given these overall contradictory data, future clinical trials are needed to assess further if an anti-VEGF therapies sustainably affect RGC survival and thus the RNFL thickness in patients.

Here, we provide further clinical evidence for this deleterious effect of anti-VEGF therapy, but in a glaucomatous population with already compromised RGCs. Repeated injection of anti-VEGF for AMD or DME induced a significant reduction of RNFL thickness over a period of 12 months in these glaucomatous patients. These results strongly suggest that VEGF is a major autocrine factor for adult human RGCs. Further studies are needed to demonstrate the potential therapeutic interest of VEGF receptor agonists in retinal diseases.

## Materials and methods

### Further details of methods are provided in Supplement

#### Primary cell cultures from adult rat retinas

Cell culture experiments were performed with retina isolated from 8-week-old Long-Evans rats, purchased from Elevage Janvier (France). Animals were housed in the same room of animal facility under controlled conditions (21 ± 1 °C, 12 h/12 h light/dark cycles, light on at 8 a.m., and food and water available ad libitum). All experiments were in accordance with the European Union Directive (2010/63/EU) and with the ARVO (Association for Research in Vision and Ophthalmology) statement for the use of animals in ophthalmic and visual Research. Experimental protocols were approved by the local ethics committee and the Ministère de la Recherche (permission number #A-75-1739 for NF).

Mixed retinal cultures were maintained until confluence (10–12 DIV) and then used to prepare conditioned medium from retinal mixed cultures (CM-M). Primary cultures of RGCs were isolated using an immunopanning technique, according to the protocol adapted from that used on adult rats^43,44^. Purified RGCs were seeded at various densities and cultured in Neurobasal-A medium (Invitrogen, Carlsbad, CA, USA) containing glutamine and supplemented with B27 (Invitrogen).

### Mesenchymal stem cell (MSC) collection

Bone marrow was obtained from the femur cavities of rats after flushing with minimal essential medium (α-MEM, Invitrogen) containing 10% fetal calf serum. MSCs were seeded at 200,000 cells/cm^2^ and cultured until confluence.

### Cell treatments

Enriched RGC were subjected to different treatment during the whole period of the RGC cultures (6 DIV). Conditioned media were applied at the dilution of 1:2 directly into RGC culture medium. The monoclonal mouse anti-VEGF-A (anti-pan against all VEGF-A isoforms, Santa-Cruz Biotechnology, Dallas, TX, USA), the polyclonal rabbit anti-murine VEGF-A_164_ (Abcam, Cambridge, UK), ranibizumab (Lucentis®, Novartis, Bâle, Switzerland; gift from Pr Matonti), a Fab fragment of the humanized monoclonal VEGF antibody (neutralizes all VEGF isoforms) and a polyclonal Ig-G (rabbit polyclonal anti-NF200; Sigma-Aldrich, Saint-Louis, MO, USA) were incubated directly into the culture medium of RGC cultures. Recombinant rat VEGF-A_164_ protein (R&D systems, Minneapolis, MI, USA), recombinant mouse VEGF-B_167_ protein (R&D systems) and recombinant rat VEGF-D_164_ protein (R&D systems) were also directly added into the medium. The antagonist at VEGF-R2 receptor, ZM 323881 (Tocris Biosciences, Ellisville, MO, USA) was co-incubated with VEGF-A_164_. ZM 323881 (5-[[7-(benzyloxy) quinazolin-4-yl]amino]-4-fluoro-2-methylphenol) is a potent and selective inhibitor of VEGF-R2 tyrosine kinase in vitro (IC_50_ < 2 nM), compared with other receptor tyrosine kinases, including VEGF-R1, a range of other receptor tyrosine kinases such as PDGFRβ, FGFR1, EGFR and erbB2 (IC_50_ > 50 µM)^45^.

### Automated counting of viable RGCs

RGC counting was realized after 6 DIV on alive cells labeled with calcein-AM (producing a green fluorescence; Invitrogen). Briefly, RGGs cultured in 96-well plate, were incubated in a calcein-AM solution (1.3 µg/ml, diluted in Neurobasal-A medium) for 1 h into the incubator (humidified chamber, 37 °C, 5%CO_2_). Calcein-positive RCGs were then automatically counted after a frame per frame scanning of the full well surface (objective X10), by using an inverted epifluorescence microscope (TiE, Nikon, Champigny-sur-Marne, France) equipped with a motorized stage and a CCD camera (Roper Scientific, Evry, France).

### Measurement of growth factors

A microparticle bead-based multiplex assay was used to measure the growth factors VEGF, BDNF, G-CSF, and GM-CSF in supernatants from mixed retinal cells, using the Luminex 200 technology. VEGF-A_164_ was specifically measured in supernatants from cell cultures using ELISA technique. Supernatants were harvested from mixed, glial or microglial or RGC cultures and frozen at − 80 °C until use. VEGF-A164 was then measured in supernatants using the “Rat VEGF Quantikine ELISA Kit” (R&D system) according the instructions of the kit.

### Patients and clinical examinations

We conducted a prospective single-center real-life study on 20 patients (10 non-glaucomatous and 10 stabilized glaucomatous patients) treated by intravitreal injections (IVI) of VEGF traps (ranibizumab or aflibercept; at least six intravitreal injections—6.4 vs 6.2—during 12 months). Patient characteristics are summarized in Table 2. The eyes receiving IVI were included in the "injected" group, whereas the follow eyes (without IVI) were included in the "non-injected" group. Informed consent was obtained from all patients in agreement with the Declaration of Helsinki for research involving human subjects.

**Table 2:** Patient characteristics.

	Age (mean ± S.D.)	Gender (% female)	Glaucoma treatments* (numbers of drugs; mean ± S.D)
Glaucoma group (n = 10)	73.8 ± 7.9	60	1.8 ± 0.6
Non glaucoma group (n = 10)	73.0 ± 9.0	70	–

*Glaucomatous patients received anti-glaucomatous treatment through eye-drop consisting in either monotherapy, bi-therapy, tri-therapy, or quadri-therapy with drugs from the 4 distinct classes (Beta blockers, Alpha-adrenergic agonists, Prostaglandins or Carbonic anhydrase inhibitors).

Non glaucomatous patients did not receive any eye treatment.

This study did not include a placebo group because it consists in a real-life study, and not a randomized, placebo-controlled and double-blind trial.

The study was approved by the Ethics Committee of the French Society of Ophthalmology (IRB 00008855 Société Française d’Ophtalmologie IRB#1).

### Statistics

All data are expressed as means ± SEM. In ELISA experiments for detection of VEGF in culture medium, undetectable values (below the detection threshold) were defined as zero and included in the mean calculations.

The Mann Whitney test was used to compare means of 2 groups. When more than 2 groups were compared, a non-parametric Kruskal–Wallis analysis of variance was performed and followed, in case of significance by a Dunns post-hoc test to compared means between each group. For clinical observations, the Friedman test was used for analysis of variance for repeated measures and followed by a Dunns *post-hoc* test to compared means between each group. For all statistical analysis, differences were considered significant at *p ≤ 0.05, **p ≤ 0.01 and ***p ≤ 0.001.

## Supplementary information

Supplementary information

## References

[1] Leung DW, Cachianes G, Kuang WJ, Goeddel DV, Ferrara N (1989). Vascular endothelial growth factor is a secreted angiogenic mitogen. Science.

[2] Ferrara N, Gerber HP, LeCouter J (2003). The biology of VEGF and its receptors. Nat Med.

[3] Otrock ZK, Makarem JA, Shamseddine AI (2007). Vascular endothelial growth factor family of ligands and receptors: review. Blood Cells Mol Dis.

[4] Ruiz de Almodovar C, Lambrechts D, Mazzone M, Carmeliet P (2009). Role and therapeutic potential of VEGF in the nervous system. Physiol. Rev..

[5] Matsuzaki H (2001). Vascular endothelial growth factor rescues hippocampal neurons from glutamate-induced toxicity: signal transduction cascades. FASEB J..

[6] Svensson B (2002). Vascular endothelial growth factor protects cultured rat hippocampal neurons against hypoxic injury via an antiexcitotoxic, caspase-independent mechanism. J. Cereb. Blood Flow Metab..

[7] Suzuki M (2011). Neuroprotective response after photodynamic therapy: role of vascular endothelial growth factor. J Neuroinflamm..

[8] Ford KM, Saint-Geniez M, Walshe T, Zahr A, D'Amore PA (2011). Expression and role of VEGF in the adult retinal pigment epithelium. Invest. Ophthalmol. Vis. Sci..

[9] Saint-Geniez M (2008). Endogenous VEGF is required for visual function: evidence for a survival role on muller cells and photoreceptors. PLoS ONE.

[10] Bird AC (2010). Therapeutic targets in age-related macular disease. J. Clin. Investig..

[11] Avery RL (2006). Intravitreal bevacizumab (Avastin) for neovascular age-related macular degeneration. Ophthalmology.

[12] Rosenfeld PJ (2006). Ranibizumab for neovascular age-related macular degeneration. N. Engl. J. Med..

[13] Martinez-de-la-Casa JM (2012). Retinal nerve fiber layer thickness changes in patients with age-related macular degeneration treated with intravitreal ranibizumab. Invest. Ophthalmol. Vis. Sci..

[14] Nishijima K (2007). Vascular endothelial growth factor-A is a survival factor for retinal neurons and a critical neuroprotectant during the adaptive response to ischemic injury. Am. J. Pathol..

[15] Kilic U (2006). Human vascular endothelial growth factor protects axotomized retinal ganglion cells in vivo by activating ERK-1/2 and Akt pathways. J. Neurosci..

[16] Foxton RH (2013). VEGF-A is necessary and sufficient for retinal neuroprotection in models of experimental glaucoma. Am. J. Pathol..

[17] Fuchs C (2005). Retinal-cell-conditioned medium prevents TNF-alpha-induced apoptosis of purified ganglion cells. Invest. Ophthalmol. Vis. Sci..

[18] Eichler W, Kuhrt H, Hoffmann S, Wiedemann P, Reichenbach A (2000). VEGF release by retinal glia depends on both oxygen and glucose supply. NeuroReport.

[19] Robbins SG, Conaway JR, Ford BL, Roberto KA, Penn JS (1997). Detection of vascular endothelial growth factor (VEGF) protein in vascular and non-vascular cells of the normal and oxygen-injured rat retina. Growth Factors.

[20] Rao S (2013). A direct and melanopsin-dependent fetal light response regulates mouse eye development. Nature.

[21] implications in neurodevelopment and neurodegeneration (2013). Carmeliet, P. & Ruiz de Almodovar, C. VEGF ligands and receptors. Cell. Mol. Life Sci. CMLS.

[22] Lee JJ (2012). High-mobility group box 1 protein is implicated in advanced glycation end products-induced vascular endothelial growth factor A production in the rat retinal ganglion cell line RGC-5. Mol. Vis..

[23] Ogunshola OO (2002). Paracrine and autocrine functions of neuronal vascular endothelial growth factor (VEGF) in the central nervous system. J. Biol. Chem..

[24] Van Den Bosch L (2004). Effects of vascular endothelial growth factor (VEGF) on motor neuron degeneration. Neurobiol. Dis..

[25] Saint-Geniez M, Maldonado AE, D'Amore PA (2006). VEGF expression and receptor activation in the choroid during development and in the adult. Invest. Ophthalmol. Vis. Sci..

[26] Famiglietti EV (2003). Immunocytochemical localization of vascular endothelial growth factor in neurons and glial cells of human retina. Brain Res..

[27] Roubeix C (2015). Intraocular pressure reduction and neuroprotection conferred by bone marrow-derived mesenchymal stem cells in an animal model of glaucoma. Stem Cell Res. Therapy.

[28] Zhao T (2011). Protective effects of human umbilical cord blood stem cell intravitreal transplantation against optic nerve injury in rats. Graefe's archive for clinical and experimental ophthalmology = Albrecht von Graefes Archiv fur klinische und experimentelle Ophthalmologie.

[29] Li Y (2008). VEGF-B inhibits apoptosis via VEGFR-1-mediated suppression of the expression of BH3-only protein genes in mice and rats. J. Clin. Investig..

[30] Skold MK, Risling M, Holmin S (2006). Inhibition of vascular endothelial growth factor receptor 2 activity in experimental brain contusions aggravates injury outcome and leads to early increased neuronal and glial degeneration. Eur J Neurosci.

[31] Deng J, Wu DZ, Gao R (1999). Elevated vascular endothelial growth factor levels in the vitreous of patients with proliferative diabetic retinopathy. Yan Ke Xue Bao.

[32] Hernandez C (2001). Vitreous levels of vascular cell adhesion molecule and vascular endothelial growth factor in patients with proliferative diabetic retinopathy: a case-control study. Diabetes Care.

[33] Sawada O, Kawamura H, Kakinoki M, Sawada T, Ohji M (2007). Vascular endothelial growth factor in aqueous humor before and after intravitreal injection of bevacizumab in eyes with diabetic retinopathy. Arch. Ophthalmol..

[34] Flammer J (2013). The eye and the heart. Eur. Heart J.

[35] Sun C (2020). Angiogenic and inflammatory biomarker levels in aqueous humor and vitreous of neovascular glaucoma and proliferative diabetic retinopathy. Int. Ophthalmol..

[36] Watanabe D (2005). Vitreous levels of angiopoietin 2 and vascular endothelial growth factor in patients with proliferative diabetic retinopathy. Am. J. Ophthalmol..

[37] Marie M (2019). Blue-violet light decreases VEGFa production in an in vitro model of AMD. PLoS ONE.

[38] Beck M, Munk MR, Ebneter A, Wolf S, Zinkernagel MS (2016). Retinal ganglion cell layer change in patients treated with anti-VEGF for neovascular age related macular degeneration. Am. J. Ophthalmol..

[39] Demirel S, Batioglu F, Ozmert E, Erenler F (2015). The effect of multiple injections of ranibizumab on retinal nerve fiber layer thickness in patients with age-related macular degeneration. Curr. Eye Res..

[40] Horsley MB, Mandava N, Maycotte MA, Kahook MY (2010). Retinal nerve fiber layer thickness in patients receiving chronic anti-vascular endothelial growth factor therapy. Am. J. Ophthalmol..

[41] Sobaci G, Gungor R, Ozge G (2013). Effects of multiple intravitreal anti-VEGF injections on retinal nerve fiber layer and intraocular pressure: a comparative clinical study. Int. J. Ophthalmol..

[42] Saleh R, Karpe A, Zinkernagel MS, Munk MR (2017). Inner retinal layer change in glaucoma patients receiving anti-VEGF for neovascular age related macular degeneration. Graefe's archive for clinical and experimental ophthalmology = Albrecht von Graefes Archiv fur klinische und experimentelle Ophthalmologie.

[43] Barres BA, Silverstein BE, Corey DP, Chun LL (1988). Immunological, morphological, and electrophysiological variation among retinal ganglion cells purified by panning. Neuron.

[44] Froger N (2012). Taurine provides neuroprotection against retinal ganglion cell degeneration. PLoS ONE.

[45] Whittles CE (2002). ZM323881, a novel inhibitor of vascular endothelial growth factor-receptor-2 tyrosine kinase activity. Microcirculation.

