# Supplementary material for: VEGF is an autocrine/paracrine neuroprotective factor for injured retinal ganglion neurons
Source: Sci Rep. 2020 Jul 24;10:12409. doi: 10.1038/s41598-020-68488-z (PMC7382485; doi:10.1038/s41598-020-68488-z)
Supplement: Supplementary file 1 — Supplementary information [file 41598_2020_68488_MOESM1_ESM.docx]

**VEGF is an autocrine/paracrine neuroprotective factor for injured retinal ganglion neurons**

Nicolas Froger^1^, Frédéric Matonti^1^, Christophe Roubeix^1^, Valérie Forster^1^, Ivana Ivkovic^1^, Nadège Brunel^2^, Christophe Baudouin^1,3^, José-Alain Sahel^1,3,4,5^ and Serge Picaud^1,4^

^1^Sorbonne Université, INSERM, CNRS, Institut de la Vision, 75012 Paris, France

^2^UMS 29 INSERM plateforme FluExGen UPMC, 75012 Paris, France

^3^CHNO des Quinze-Vingts, DHU Sight Restore, INSERM-DGOS CIC 1423, 75012 Paris, France

^4^Fondation Ophtalmologique Adolphe de Rothschild, 75020 Paris, France

^5^Department of Ophthalmology, The University of Pittsburgh School of Medicine, Pittsburgh, PA 15213, United States

**----------------------------------------**

**Supplemental Information**

**I- Supplemental results**

***I-1. Conditioned medium from retinal mixed cultures promotes RGC survival***

We previously showed that conditioned medium produced by mixed rat retinal cells (CM-M) can promote survival of purified rat RGCs ([Fuchs et al., 2005](#_ENREF_4)). Under serum-deprivation (B27-free medium, low-nutritive condition), a low density of calcein-positive RGCs was found after 6 DIV (5.9 ± 1.9 cells per field, mean±SEM; Fig. S1A, S1E). Additional 93% of the cells died in the following 3 days (9 DIV, Fig. S1E), while almost all RGCs being dead at 15 DIV (Fig. S1C, S1E). Incubation of the CM-M enhanced significantly the neuronal survival at 6 DIV (+191% survival; p<0.05), 9 DIV (+260% survival; p<0.05) and 15 DIV (+700% survival; p<0.05) as compared to RGC cultured in control condition (Fig. S1B, D & S1E).

A similar trend was observed when the CM-M was prepared with medium enriched with the B27 supplement (high nutritive condition), but the rate of RGC degeneration was significantly decreased. For instance, the presence of B27 supplement in the control medium increased by ~2.3 fold the RGC survival at 6 DIV (Fig. S1F) and some RGCs can survive up to 35 DIV. Again, the B27-containing CM-M enhanced significantly RGC survival from 6 DIV (Fig. S1F, G & S1J), until 21 DIV (Fig. S1H, I & S1J). All thereafter experiments were performed with pure B27-free medium.


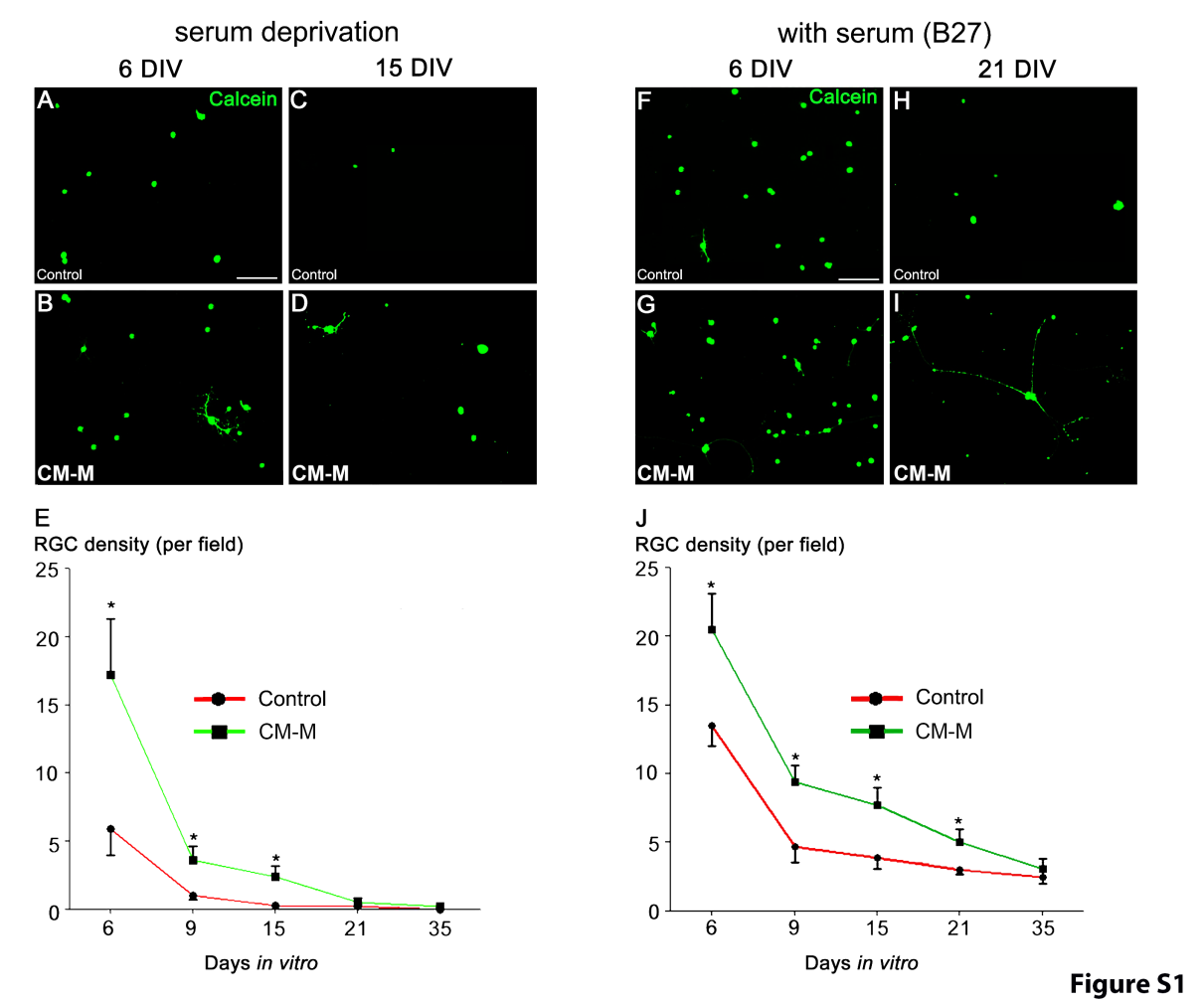


**Figure S1:** **Conditioned medium harvested from retinal mixed cultures sustainably enhanced the RGC survival.**

A-D: Representative fluorescence images of calcein-positive alive RGCs cultured in low nutritive conditions (serum deprivation), after 6 DIV (A, B) or 15 DIV (C, D), either in control medium (Control; A, C) or in the presence of conditioned medium from retinal mixed cultures (CM-M; B, D).

E: Kinetics of alive RGCs densities achieved either in control medium (Control; red line) or in presence of conditioned medium (CM-M; green line). Data, expressed as number of RGC per field, are means±SEM from independent cultures (n=6-10). *p<0.05 as compared to control conditions, at each DIV considered (Mann-Whitney test).

F-I: Representative fluorescence images of calcein-positive RGCs after 6 DIV (F, G) or 21 DIV (H, I) cultured in high nutritive conditions (with B27; F, H) either in control medium (F, H or in the conditioned medium from retinal mixed cultures (CM-M; G, I).

J: Kinetics of alive RGG densities achieved either in control medium (control, red line) or in conditioned medium (CM-M, green line). Data, expressed as number of RGC per field, are means ± SEM from independent measures (n=4-8). *p<0.05 as compared to control at each DIV considered (Mann-Whitney test).

***I-2. Purified microglial cells produce VEGF.***

In order to identify the cellular source(s) of the VEGF production from the retinal mixed culture, we labeled the different cell types that may be contained in the culture (Fig. S2A). Apart from retinal neurons, such as peanut lectin-stained cone photoreceptors (Fig. S2E), G_0α_-immunopositive ON bipolar cells (Fig. S2D) and few Brn-3a-immunopositive retinal ganglion cells (Fig. S2F), the mixed retinal cultures contained a monolayer of vimentin- or GFAP-immunopositive macroglial cells (Fig. S2B) and OX42-immunopositive microglial cells (Fig. S2C).


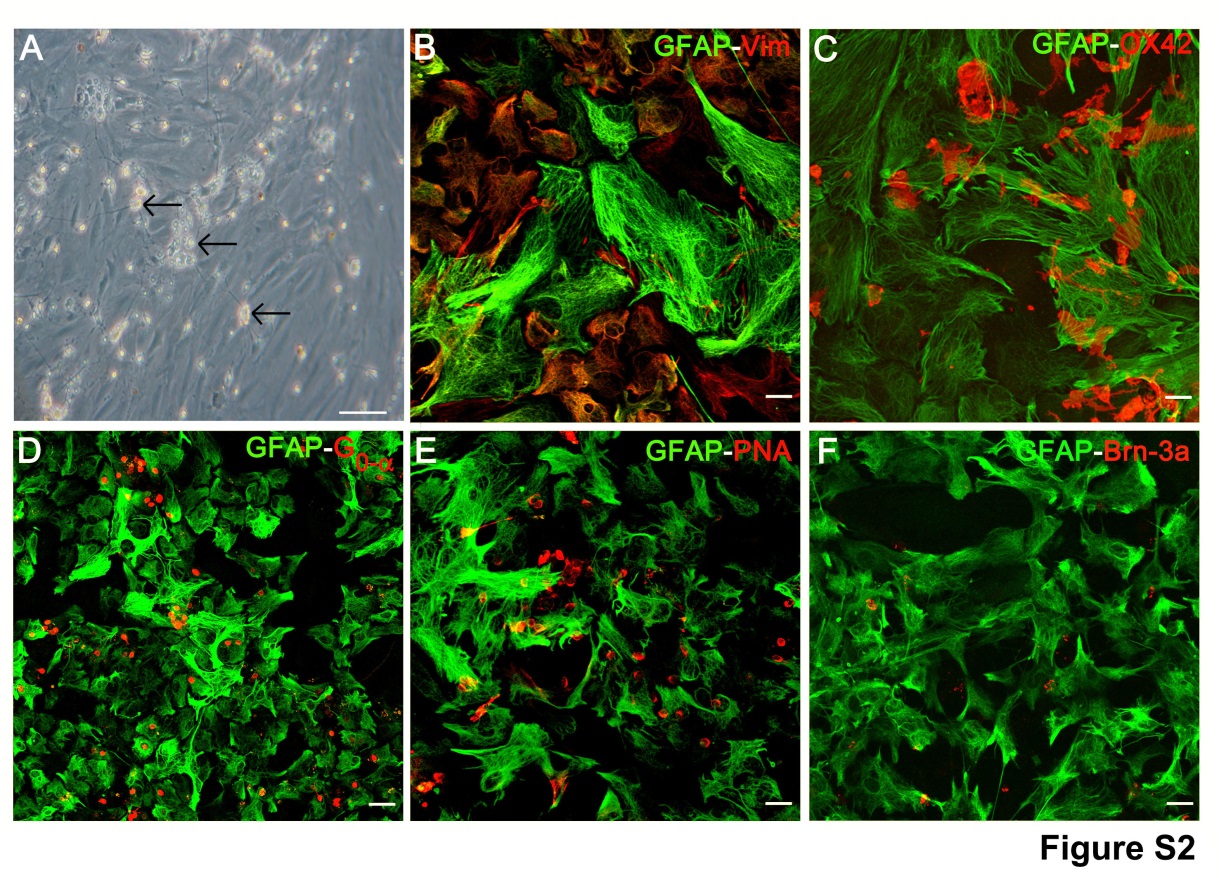


**Figure S2:** **Characterization of cellular types which compose the retinal mixed cultures**

A: Representative bright field image showing a confluent retinal mixed culture at 10-12 DIV. Cultures were constituted by differentiated neuronal cells growing upon a glial monolayer. Scale bar represents 50 µm.

B-C: Representative confocal images showing the immunofluorescence labeling of glial cells in retinal mixed cultures. Cells were co-labelled with GFAP (for astrocytes, in green) and vimentin (for Müller cells, in red; B) or OX42 (for microglia, in red; C), respectively. Note the high density of microglia in C. Scale bar represents 20 µm.

D-F: Representative confocal images showing the immunofluorescence labelling of neuronal cells in retinal mixed cultures. Mixed cultures were co-labelled with GFAP (for glial cells, in green; D-F) and G_0α_ (for bipolar cells, in red; D), or PNA (for cone photoreceptor, in red; E) or Brn-3a (for RGCs, in red; F), respectively. Scale bar represents 20 µm.

Representative panels from 3 independent mixed cultures

Isolated Muller cells (Eichler et al., 2000) or a human Müller cell line (MIO-M1) (Saint-Geniez et al., 2008) were previously reported to produce VEGF. We therefore prepared a culture of enriched Müller cells to collect the conditioned medium. Note that the purity of these macroglial cell cultures was validated by the absence of G_0-α_-positive bipolar cells (Fig S3A). Surprisingly, conditioned medium from these macroglial cells (CM-G) did not generate VEGF-A_164_ except in one case at the detection threshold (16 pg/ml; Fig. S3C).

By contrast, cultures of purified microglial cells generated substantial levels of VEGF (17.8 ± 4.6 pg/ml; mean ± SEM with undetectable concentrations were defined as zero; Fig. S3C), as detected in conditioned medium from microglial cells (CM-MG) in majority of cases (55%) after only 48h *in vitro* (Fig. S3C). The purity of microglial cell cultures was estimated at 91.9% ± 0.1 (mean ± SEM, n=3), as indicated by OX-42-immunodection (Fig. S3B). These results suggested that VEGF released by mixed retinal cells in the CM-M can originate from the microglial cells contained in the culture.


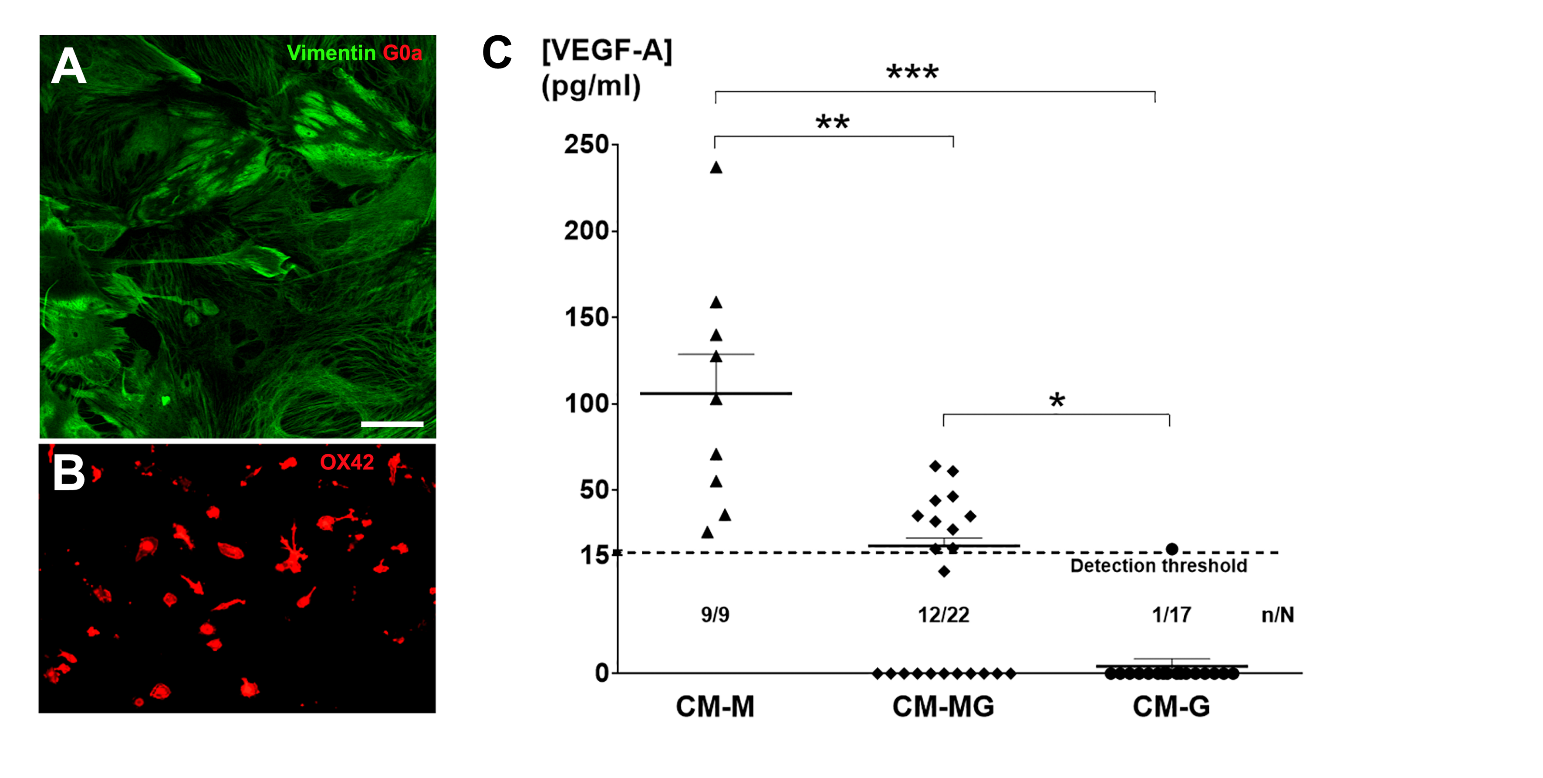


**Figure S3:** **VEGF is produced by retinal microglial cultures but undetectable in retinal glial cultures**

A-B: Confocal images showing the immunofluorescence staining of enriched retinal glial cultures (A). Glial cells were labelled with vimentin (in green), whereas bipolar cells, labelled with G_0α_, are absent. B: Immunofluorescence staining of pure retinal microglial cultures. Microglial cells were labelled with the specific marker OX42. Representative panels from 3 independent cultures; scale bar represents 50µm

C: VEGF-A_164_ amounts measured by ELISA, in conditioned medium from mixed retinal cell cultures (CM-M), in conditioned medium from retinal glial cultures (CM-G) and finally in conditioned medium from retinal microglial cultures (CM-MG). Note that the majority of conditioned media from microglial cultures showed the presence of detectable VEGF amounts, whereas in conditioned media from glial cultures VEGF amounts were found undetectable (except for one sample).

Data, expressed as pg/ml, are means ± SEM from independent cultures, and n/N refers to the number of conditioned media showing detectable amounts of VEGF-A_164_ as compared to the total number of conditioned media tested. *p<0.05, **p<0.01 and ***p<0.001 as compared to the indicated groups (

**I-3. VEGF is expressed by RGCs**

To further assess the presence of VEGF in cultured RGCs, we performed an immunolabeling with anti VEGF on pure RGCs after 6 DIV (Fig. S4A-C). Although RGC had been purified prior to the culture, we also controlled their identity using the β-III tubulin specific marker. All cells were β-III tubulin positive (Fig. S4B), and counterstained with DAPI (Fig. S4D). They also displayed a positive immunostaining with the anti-VEGF_164_ (Fig S4A), which appeared rather localized in RGC somas (Fig S4C).

In order to define if RGCs also contained VEGF *in situ*, we immunolabeled rat retinal sections with the anti-VEGF_164_ antibody while identifying RGCs using the Brn-3a specific marker localized in RGC nuclei (Fig. S4F, H). RGCs were found strongly VEGF immunopositive, whereas cells of the inner nuclear layer (INL) were not immunolabeled (Fig S4D-F). These results indicated that RGCs can express VEGF protein both *in vitro* and *in situ*. Finally, we addressed the presence of VEGF mRNA in RGCs by RT-PCR experiments. We well found the presence of specific mRNA from VEGF-A gene in purified RGCs (data not shown).


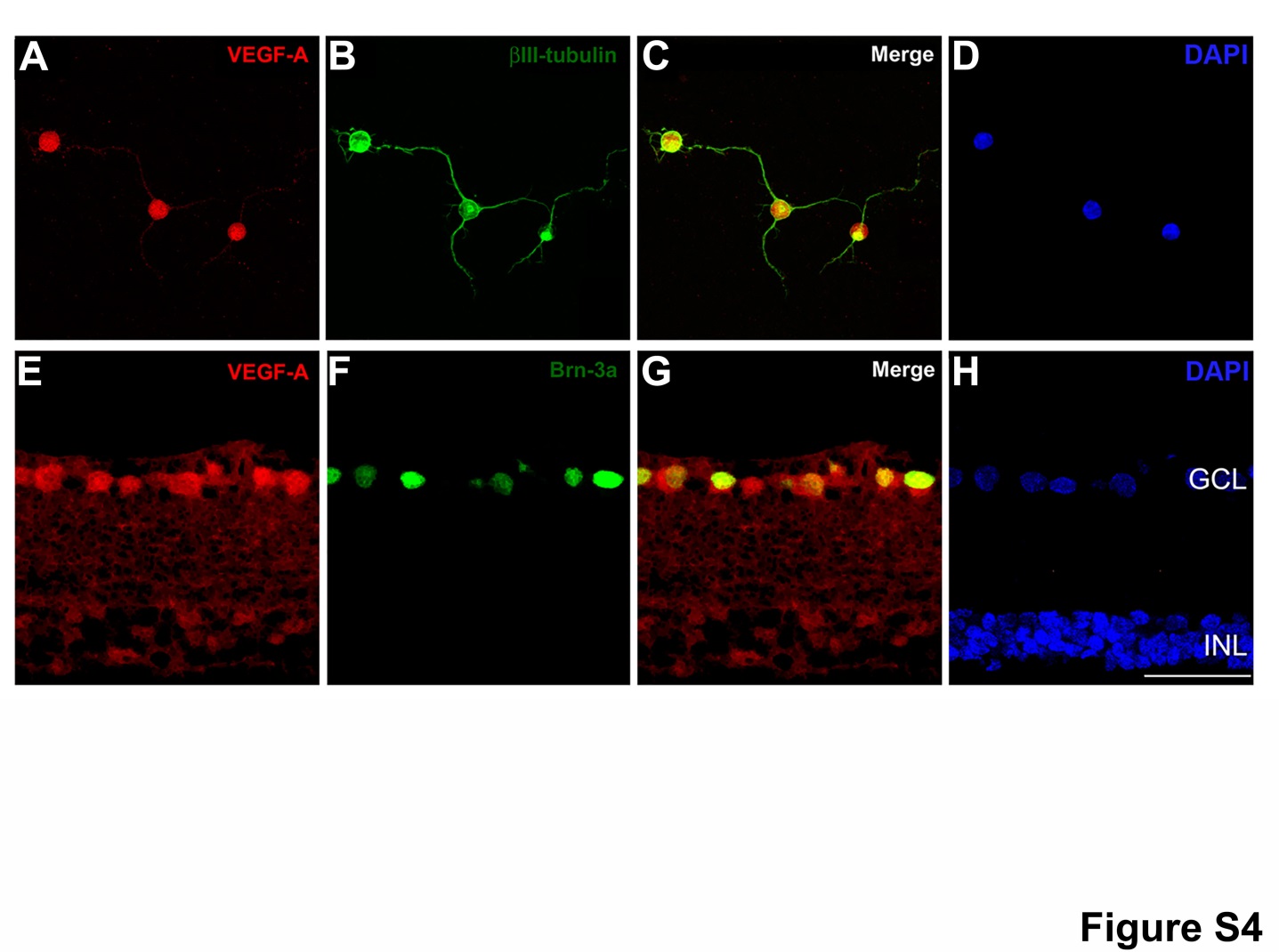


**Figure S4:** **VEGF is expressed by RGCs in culture and *in situ* retinal sections**

A-D: Representative confocal images showing the VEGF expression in cultured RGCs after 6 DIV (in red, A) when labelled with the anti-VEGF-A_164_. Cultured RGCs were co-labelled with the anti-β-III tubulin specific marker (in green, B) and counterstained with DAPI to visualize the cell nuclei (in blue, D).

D-H: Representative images showing of VEGF expression in the ganglion cell layer (GCL) of retinal sections labelled with the anti-VEGF-A_164_ (in red, E). RGCs were specifically labelled with the anti-Brn-3a (in green, F) and counterstained with DAPI to visualize the cell nuclei (in blue, H).

Scale bar: 50 µm.

**II- Detailed description of methods**

***II-1. Primary cell cultures from adult rat retinas***

*II-1.1. Mixed cultures from whole retinas*

Primary mixed cultures were firstly described by Gaudin et al ([Gaudin et al., 1996](#_ENREF_5)), and then slightly modified by Fuchs *et al*. ([Fuchs et al., 2005](#_ENREF_4)). Three Long Evans rats were anesthetized and killed; their eyes were removed and rinsed in the CO_2_-independent culture medium (Invitrogen, Carlsbad, CA, USA). Retinas were then dissected in the same solution, under a microscope. Isolated retinas were chopped into small fragments and washed in Ringer’s solution containing (in mM): NaCl 125; KCl 3.6; MgCl_2_ (6H_2_0) 1.18; NaHCO_3_ 22.6; NaH_2_PO_4_ 0.02; Na_2_HPO_4_ 0.028; Na_2_SO_4_ 1.2; Glucose 10 and supplemented with 0.54 mM EDTA(Na_2_) (Sigma-Aldrich St-Louis, MO, USA, for all products). They then were incubated in papain solution (4 UI/mL; Worthington, Lakewood, NJ, USA) in the same buffer for 25 minutes at 37°C. Previously, papain needed to be activated for 30 min in a solution containing (in mM) EDTA 70; L-cystein 275 and β-mercaptoethanol 3.72 (Sigma-Aldrich for all products). After digestion, papaïn activity was stopped by addition of DMEM (Invitrogen) supplemented with 10% fetal bovine serum (FBS, Invitrogen), containing 50 UI of DNAse (Sigma-Aldrich). Tissues then were immediately dissociated by repeated gentle triturating in 4-5 steps. Cell suspension was centrifuged at 800 rpm for 5 min. The supernatant was removed, and cells were resuspended in DMEM+10% FBS. Cells were seeded into one 6-well plate (Corning, NY, USA) for six retinas (which corresponds to a seeding density at 15.8x10^6^±1.9x10^6^ cells/well; mean ± SEM, n=4), containing coverslips (for future immunocytochemistry), previously coated with poly-D-lysine (2µg/cm^2^; for one hour) and laminin (1µg/cm^2^; overnight; both purchased from Sigma-Aldrich). Cells were finally incubated at 37°C in a humidified atmosphere of 5% CO_2_-95% air. Culture medium was changes one time at 3 days *in vitro* (DIV), and cultures were kept until confluence (10-12 DIV). To prepare conditioned medium from mixed cultures (CM-M), cells were rinsed and incubated with Neurobasal-A medium containing glutamine. After 48h incubation, the supernatants were harvested, centrifuged for 10 min at 200 g, +4°C, and finally aliquoted at -80°C until use.

*II-1.2. Retinal glial cultures*

Cultures of retinal glia were obtained by seeding a similar cell suspension as that prepared for mixed cultures, at the same density (*i.e*. 6 retinas for one 6-well plate). After 48h, the culture medium (DMEM + 10% FBS) was changed. Cells were vigorously rinsed to detach neurons, which are less adhered than glial cells. After 10-12 DIV, supernatants from confluent cultures, exclusively composed by a glial monolayer, were harvested and frozen as conditioned medium from glial retinal cells (CM-G).

*II-1.3. Retinal microglia cultures*

Cultures of retinal microglia were also obtained with the similar cell suspension as prepared for mixed cultures. After one change of medium at 3 DIV, confluent mixed cultures (12-14 DIV) were gently shacked to obtain a microglial cell suspension. Supernatants from one 6-well plate were then collected and replace in one 35-mm dish containing glass coverslips (for future immunocytochemistry). After 48h incubation in the culture medium (DMEM+10%FBS), microglial cells were adhered to the glass coverslips. Supernants from pure microglial cultures were then harvested and frozen as conditioned medium from microglial retinal cells (CM-MG).

*II-1.4. Pure retinal ganglion cell (RGC) cultures*

Primary cultures of RGCs were isolated from retinas of adult Long-Evans rat with an immunopanning technique, according to the protocol previously described in young rats by Barres et al. (1988) ([Barres et al., 1988](#_ENREF_1)) and subsequently adapted on adult rats ([Froger et al., 2012](#_ENREF_3)). Six rats were anesthetized and killed by cerebral dislocation. Their eyes were removed and placed in a solution of phosphate-buffered saline (PBS) containing 1 g/L of glucose (PBS-glucose; Invitrogen, Carlsbad, CA, USA). The twelve retinae were isolated and rinsed in PBS-glucose and incubated in this same buffer containing 33 UI/mL of papaïn (Worthington, Lakewood, NJ, USA) and 200 UI/mL of DNAse (Sigma-Aldrich, St-Louis, MO, USA) for 30 min at 37°C. They were then rinsed in PBS-glucose, containing 0.15% ovomucoid (Roche Diagnosis, Basel, Switzerland) and 0.15% bovine serum albumin (BSA; Sigma-Aldrich) to stop the enzymatic reaction. Retinas were dissociated in this same latter buffer, supplemented with 333 UI/mL of DNAse and a rabbit anti-rat macrophage (5 mg/ml; Accurate Chemical & Scientific Corporation, Westbury, NY, USA) in three steps, using pipettes with decreasing tip diameters. The cell suspension was centrifuged at 115 g during 13 min at room temperature. The supernatant was removed, and cells were suspended in PBS-glucose, containing 1% ovomucoid and 1% BSA. After a second centrifugation (115 g, 13 min), cells were suspended in the PBS-glucose, containing 0.02% BSA. Cell suspension was filtrated using a Sefar Nitrex mesh (48µm, Dutscher, Brumath, France) and then incubated in a dish (Ø 150 mm), previously coated with a goat anti-rabbit IgG (Jackson Immunoresearch, West Grove, PA, USA), during 36 min at room temperature. After a vigorous shaking of the dish, the cell suspension was moved into a second dish (Ø 150 mm), previously coated with the same antibody, and incubated during 33min at room temperature. After another vigorous shaking, the remaining cell suspension was transferred into a dish (Ø 100 mm), previously coated successively with (i) a goat anti-mouse IgM (Jackson Immunoresearch, West Grove, PA, USA) and (ii) a medium containing a mouse anti-Thy-1 antibody, previously prepared in our laboratory from a T11D7 hybridoma cell line (ATCC, Manassas, VA, USA). After 45 min incubation, the dish was rinsed ten times with PSB-glucose. Adherent cells remaining into the dish were RGC specifically selected by the mouse anti-Thy-1 antibody. Cells were incubated with Earle’s Balanced Salts Solution (EBSS; Sigma-Aldrich) containing 0.125% of trypsin (Sigma-Aldrich) for 10 min at 37°C, in humidified atmosphere (5% CO_2_). Trypsin action was blocked by adding PBS-glucose containing 30% inactive fetal bovine serum (FBS; Invitrogen) in the Earle’s solution. Cells were detached by 10 successive pipette flows with the mixed Earle-PBS solution, and the resulting cell suspension was centrifuged at 115 g for 15 min. Pure RGCs were then suspended in Neurobasal-A (Invitrogen) supplemented with 2mM L-glutamine (Invitrogen). As previously described RGC purity was estimated between 92% and 98% ([Froger et al., 2012](#_ENREF_3)). Pure RGCs were cultured in 96-well plates and kept in a humidified chamber at 37°C containing 5% CO_2_.

*II-1.5. Mesenchymal stem cell (MSC) collection*

Bone marrow was obtained from femur cavities from rats after flushing in minimal essential medium (α-MEM) containing 10% fetal calf serum (FCS). Cells were incubated (200,000 cells/cm2) at 37°C in 5% CO2 humidified air. No adherent cells were discarded after 72h and MSC were then routinely cultured. At near confluence, from passage 4 to 6, the cell monolayer was abundantly washed with PBS before adding Neurobasal-A medium containing glutamine for 24 h.

***II-2. Measurement of growth factors by Luminex technology***

A microparticle bead-based multiplex assay was used to measure growth (VEGF, BDNF, G-CSF and GM-CSF) in samples, as previously described. A vacuum filtration system was used for all wash steps. Standards, control or conditioned media were added into individual wells of a 96-well plate, containing VEGF-, BDNF- G-CSF- or GM-CSF-positive beads, respectively and incubated overnight at 4°C on an orbital shaker (300 rpm). After incubation, detection antibody was added to each well, and the plates were incubated for 1 hour at room temperature. After washing, the detection reagent (streptavidin-phycoerythrin) was added to each well for 30-min incubation. After washing the plates were read by a flow cytometry-based instrument (Bio-Plex Array Reader; Bio-Rad, Hercules, CA), which uses Luminex 200 (Austin, TX) technology. The mean fluorescence intensity for each well was determined as the mean fluorescence of 100 beads. The lower limit of detection was defined by the lowest standard, with fluorescence > background fluorescence + 2 SD of the background fluorescence. Analysis software (Bio-Plex Manager 5.1; Bio-Rad) converted fluorescence values to concentrations using a calibration curve derived from a five-parameter logistic fit of fluorescence readings of the standards. The upper limit of detection was determined by the highest standard, having measured concentration back-calculated from the standard curve within 70% of the expected value. The quality of the fit of the standard curve to the data was good, as judged by graphical representation and the criteria that all standards between the upper and lower limits of detection had back-calculated concentrations within 70% of expected values.

***II-4. Fluorescence Immunolabelling***

Immunolabelling experiments were performed on cells cultures (immunocytochemistry) and retinal sections (immunohistochemistry).

For fluorescence immunocytochemistry, cells grew on coverslips were fixed by incubation in paraformaldehyde 4% (w/v) for 15 min at room temperature. After one rinse in Phosphate Buffer Saline 1X (PBS), cells were treated with PBS-triton 0.1% (v/v) during 10 min at RT for permeabilization and then incubated for 1 h at RT in a saturation buffer containing PBS-triton 0.1% plus 1% (w/v) of Bovine serum albumin (BSA; Sigma-Aldrich) and 0.05% (v/v) Tween 20 (Sigma-Aldrich). Immediately after saturation, cells were incubated overnight at 4°C with the primary antibody diluted in the saturation buffer. The primary antibodies used were: the polyclonal rabbit anti-GFAP (1:500; Dako, Golstrup, Denmark) for the labeling of astroglial cells, the monoclonal mouse anti-vimentin (1:500, Abcam, Cambridge, UK) for Müller cells, the monoclonal mouse anti-OX42 (1:500; BD Pharmigen, San Diego, CA, USA) for microglia, the monoclonal anti-Brn-3a (1:100, Millipore) and the monoclonal anti-βIII-tubulin (1:500; Sigma-Aldrich) for ganglion cells, the monoclonal anti-G_0α_ (1:500, Millipore) for bipolar cells and the monoclonal anti-rhodopsin (1:500, Millipore) for rod photoreceptors. In addition, the polyclonal anti-VEGF-A_164_ (1:500, Abcam) was used to detect VEGF expression in RGC. After three rinses in PBS-0.1% triton, culture cells were incubated for 1 hour at RT with the secondary antibodies, diluted in the saturation buffer: a goat anti mouse IgG alexa fluor^®^ 594 (1:500; Invitrogen) and a goat anti-rabbit IgG Alexa fluor^®^ 488 (1:500; Invitrogen). The cone photoreceptor was labeled using the lectin PNA (from the peanut *Arachis hypogaea*) Alexa-fluor^®^ 594 conjugated (1:100; invitrogen), which was incubated instead of secondary antibody in the same time and temperature. After three rinses with PBS 1X, coverslips were mounted on glass slides, using the Permafluor^®^ reagent (Microm, Francheville, France).

For fluorescence immunohistochemistry on retinal sections, rats were anesthetized, killed by cerebral dislocation and their eyes were removed and placed into a fixative solution containing 4% formaldehyde (Merck chemicals, Darmstadt, Germany) for 24 hours. Eye cups were isolated and cryo-protected in successive solutions of PBS containing 10%, 20% and 30% sucrose at 4°C, oriented along the dorso-ventral axis and embedded in NEG50^®^ (Microm, Francheville, France), frozen in liquid nitrogen and store at -20°C, until use. Retinal sections (12μm) were performed with Cryostat (Microm HM560), placed on Superfrost microscope glass slides, and kept at -80°C until use. Retinal sections were permeabilized for five minutes in PBS containing 0.1% Triton X-100 (Sigma, St. Louis, MO), rinsed, and incubated in PBS containing 1% Bovine Serum Albumin (BSA; Sigma) and 0.05% Tween 20 (Sigma) for one 1 hour at room temperature. The primary antibodies – monoclonal anti-Brn-3a antibody (1:100; Millipore, Billerica, MA, USA) and polyclonal anti-VEGF-A_164_ (1:500, Abcam) – were added to the solution and incubated overnight at 4°C. Sections were rinsed and then incubated with the secondary antibodies, goat anti-mouse IgG AlexaFluor^®^ 594 (1:500, Invitrogen) and goat anti-rabbit IgG AlexaFluor® 488 (1:500, Invitrogen), for one hour at room temperature. To label cell nuclei, 4',6-Diamidino-2-phenylindole (DAPI;10μg/mL) were incubated with the secondary antibody. Sections were rinsed three times with PBS and mounted with Permafluor^®^ reagent (Microm, Francheville, France).

***II-5. Patients and clinical examinations***

A prospective single-center study was conducted on 20 patients treated by intravitreal injections (IVI) of anti-VEGF traps (ranibizumab or aflibercept; at least six intravitreal injections – respectively 6.4 vs 6.2 - during 12 months). Ten were glaucomatous stabilized patients and 10 were non-glaucomatous patients. The injected eyes received a ranibizumab or aflibercept loading dose (3 monthly injections) followed by a capped pro-re nata (PRN) regimen with monthly visits. The eyes receiving IVI were included in the "injected group” while the fellow eyes (not receiving IVI) were included in the "control group”. Informed consent was obtained from all patients in agreement with the Declaration of Helsinki for research involving human subjects.

*II-5.1. Inclusion criteria*

The “injected group” consisted of treated eyes with anti-VEGF IVI for either a wet Age-related macular degeneration (AMD) in both glaucomatous (n=6) and non-glaucomatous (n=6) patients, or a diabetic macular edema (DME) in both glaucomatous (n=4) and non-glaucomatous (n=4) patients. The diseases were confirmed using Spectral Domain Optical coherence tomography (SD-OCT) and fluorescein and green infracyanine angiographies (Heidelberg spectralis OCT and HRA, Heidelberg Engineering, Heidelberg, Germany). The exclusion criteria were: poor quality SD-OCT images, any active ocular disease associated (such as uveitis, infections...), patient under 18 years old and pregnancy. In the “injected group”, 18/20 patients received ranibizumab (Lucentis^®^ 0.5mg/0.05mL) while 2/20 patients aflibercept (Eylea^®^ 2mg/0.05mL) at each injection. A series of 3 IVI with a one-month interval was scheduled for inclusion in the study. Then, patients were retreated following a *pro re nata* (PRN) protocol.

The control group consisted of the fellow eye (non-injected) for each patient included. The exclusion criteria were: a history of treatment with IVI dating less than 6 months in the control eye or a treatment during the study, eyes with poor SD-OCT images, any active ocular pathology associated (such uveitis, infection ...), patient under 18 years old, pregnancy.

*II-5.2. Clinical examination*

The baseline examinations included the measurements of best corrected visual acuity (BCVA), slit lamp and fundus examinations and intra ocular pressure (IOP) measurements, using Goldmann applanation tonometry, at a fixed 1-month interval. These clinical examinations were associated with an optical coherence tomography (OCT) examination: the OCT retinal nerve fiber layer (RNFL) scans were performed at a fixed 3-months interval using the Heidelberg spectral Domain (Spectralis, Heidelberg Engineering, Germany). The circle scan is 12° in diameter, and the diameter in mm is mainly dependent of the axial length of the eye. For a classical eye length, the circle is approximately 3.5-3.6 mm in diameter.

The images were acquired using image alignment eye-tracking software (TruTrack, Heidelberg Engineering, Germany) to obtain reproducible retinal circle scans. By using the follow-up function, the exact protocol was repeated each 3 months at the same retinal location.

Baseline was defined as the visit for the first intravitreal therapy was done. For retinal segmentation, the inbuilt Heidelberg Eye Explorer (Heidelberg Engineering, Germany) was used to analyze the mean RNFL thickness.

Standard Automated Perimetry (SAP) was done using the Humphrey (Zeiss) but due to the macular disease and the lack of reliability due to the loss of fixation, the visual fields were not included in the study.

**III- References**

Barres, B.A., B.E. Silverstein, D.P. Corey, and L.L. Chun. 1988. Immunological, morphological, and electrophysiological variation among retinal ganglion cells purified by panning. *Neuron* 1:791-803.

Eichler, W., H. Kuhrt, S. Hoffmann, P. Wiedemann, and A. Reichenbach. 2000. VEGF release by retinal glia depends on both oxygen and glucose supply. *Neuroreport* 11:3533-3537.

Froger, N., L. Cadetti, H. Lorach, J. Martins, A.P. Bemelmans, E. Dubus, J. Degardin, D. Pain, V. Forster, L. Chicaud, I. Ivkovic, M. Simonutti, S. Fouquet, F. Jammoul, T. Leveillard, R. Benosman, J.A. Sahel, and S. Picaud. 2012. Taurine Provides Neuroprotection against Retinal Ganglion Cell Degeneration. *PloS one* 7:e42017.

Fuchs, C., V. Forster, E. Balse, J.A. Sahel, S. Picaud, and L.H. Tessier. 2005. Retinal-cell-conditioned medium prevents TNF-alpha-induced apoptosis of purified ganglion cells. *Investigative ophthalmology & visual science* 46:2983-2991.

Gaudin, C., V. Forster, J. Sahel, H. Dreyfus, and D. Hicks. 1996. Survival and regeneration of adult human and other mammalian photoreceptors in culture. *Investigative ophthalmology & visual science* 37:2258-2268.

Saint-Geniez, M., A.S. Maharaj, T.E. Walshe, B.A. Tucker, E. Sekiyama, T. Kurihara, D.C. Darland, M.J. Young, and P.A. D'Amore. 2008. Endogenous VEGF is required for visual function: evidence for a survival role on muller cells and photoreceptors. *PloS one* 3:e3554.
